# The Preservation of Infants in Delivery

**Published:** 1847-10-01

**Authors:** 


THE PRESERVATION OF INFANTS IN DELIVERY. Being an Exposition of the chief Cause of Mortality in Still-born Children. By *Richard King,* M.D., M.R.C.S. 8vo, pp. 60. Churchill, 1847.

IT seems this tends to prove a revolutionary era in the established usages of the practice of Midwifery. We have Dr. Simpson counselling us to abstract the placenta as a remedy for unavoidable hæmorrhage instead of turning the child. Mr. Adams tell us it is all a mistake to suppose that absence of contraction of the uterus has anything whatever to do with hæmorrhage from that organ, the blood really coming from certain hypothetical and imaginary rents and lacerations of the passages. And now we have Dr. King standing forth to protest against the error all our celebrated teachers have fallen into, in supposing that death of the still-born child results from compression of the funis during labour. On the contrary, this very pressure may become the means of saving its life, for "the frequent death of the infant in preternatural deliveries arises from syncope, and not from asphyxia; in fact, from the want of the compression of the cord, and not from the dreaded compression of it." The author's idea (another, by-the-bye, utterly at variance with all authority, and we believe all fact) is, that the after-birth is detached from the uterus simultaneously with the expulsion of the first portion of the child; when it expands, "as a moist sponge would act when released from the hand and placed in contact with water," and favours the escape of the blood from the child, who perishes from the consequent syncope. His practice is therefore, as soon as the breech is expelled, to compress the cord before the delivery of the rest of the body; and he seems even to have invented some description of forceps for the purpose !

Feeling convinced that his supposition that the placenta is detached prior to the birth of the child is only correct in some rare exceptional cases, we likewise believe that the practice he founds upon it may often prove dangerous by diverting attention from a more correct procedure, or by itself inducing an asphyxia, which otherwise might never have happened. The distinction between the asphyxia and the syncope of new-born infants Dr. King lays so much stress upon, as of novel discovery, is borne in mind by every accoucheur, at least if he allows himself to be guided by the rules laid down in our standard works.

